# Morphological and molecular characterisation of *Sarcocystis capracanis*, *Sarcocystis cornagliai* and* Sarcocystis rossii* n. sp. infecting the Alpine ibex (*Capra ibex*)

**DOI:** 10.1186/s13071-025-06737-8

**Published:** 2025-03-10

**Authors:** Eglė Rudaitytė-Lukošienė, Steffen Rehbein, Rafael Calero-Bernal, Dalius Butkauskas, Petras Prakas

**Affiliations:** 1 Nature Research Centre, Akademijos 2, 08412 Vilnius, Lithuania; 2 Formerly Kathrinenhof Research Center, 83101 Rohrdorf, Germany; 3 SALUVET Group, Animal Health Department, Complutense University of Madrid, Ciudad Universitaria s/n, 28040 Madrid, Spain

**Keywords:** *Sarcocystis*, Alpine ibex, TEM, 18S rRNA, *cox1*, Phylogeny

## Abstract

**Background:**

The cyst-forming coccidia of the genus *Sarcocystis* (Sarcocystidae) are widespread protists of mammals, particularly of domestic and wild ruminants. Research on genus *Sarcocystis* in wild members of the subfamily Caprinae is, however, rather limited. *Sarcocystis* in the Alpine ibex (*Capra ibex*) have only been investigated in depth once and then solely by morphological techniques. In the current investigation we aimed to morphologically and genetically characterise *Sarcocystis* species of Alpine ibex in Austria.

**Methods:**

Sarcocysts detected in the diaphragm and myocardium muscles were morphologically described using light microscopy and transmission electron microscopy (TEM). Isolated sarcocysts were molecularly identified and characterised at the level of the 18S ribosomal RNA (rRNA) gene and cytochrome c oxidase I gene (*cox1*). The obtained sequences were subjected to phylogenetic analysis.

**Results:**

Three *Sarcocystis* species, namely *S. capracanis*, *S. cornagliai* and *S. rossii* n. sp., were found in Alpine ibex. For the first time, we genetically characterised *S*. *cornagliai*, which is most closely related to *Sarcocystis* species that are transmitted by corvid birds. Sarcocysts of *S*. *rossii* n. sp. were found to be ribbon-shaped, with pointed tips. Hair-like protrusions about 5 μm in length were observed on sarcocyst walls. Observation of toluidine blue-stained semi-thin sections revealed that the sarcocyst of *S*. *rossii* n. sp. was thin-walled. Using TEM, cyst walls were observed to be similar to type 7a, with thin hair-like villar protrusions on the cyst wall, which were filled with many fine electron-dense granules. The ground substance layer was particularly thin, measuring 0.2–0.4 μm. The *cox1* sequences of *S*. *rossii* n. sp. had the highest similarity to those of *Sarcocystis* *arieticanis* and *Sarcocystis* *hircicanis*.* Sarcocystis rossii* n. sp. had a close phylogenetic relationship with species that use canids as definitive hosts.

**Conclusions:**

This study confirms the role of the Alpine ibex as an intermediate host of three *Sarcocystis* species and sets a new host record for *S*. *capracanis*. It also provides the first molecular data on *Sarcocystis* from Alpine ibex and on *S*. *cornagliai*. In addition, a new species, *S*. *rossii*, was identified and described. Phylogenetic analyses suggested corvid birds and canids as potential definitive hosts for *S. cornagliai* and *S. rossii* n. sp., respectively.

**Graphical Abstract:**

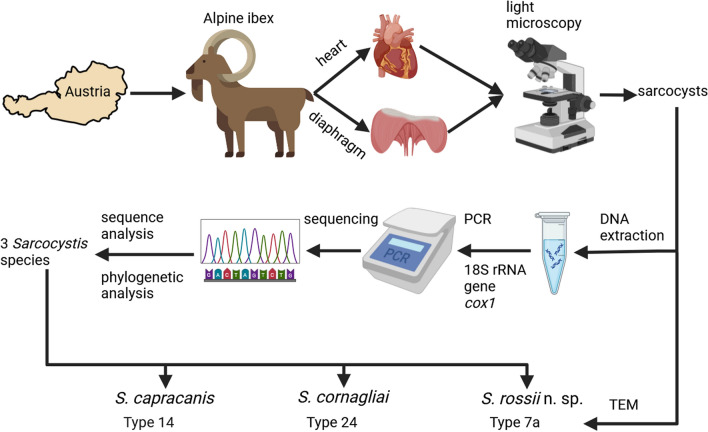

**Supplementary Information:**

The online version contains supplementary material available at 10.1186/s13071-025-06737-8.

## Background

Among the protists of the phylum Apicomplexa, the cyst-forming species of the genus *Sarcocystis* (Sarcocystidae) are widespread in mammals and notably prevalent in domestic and wild ruminants. *Sarcocystis* species are capable of causing both clinical and subclinical diseases, called sarcocystosis, in their respective hosts [1]. *Sarcocystis* spp. are characterised by a two-host life-cycle and the formation of cysts (sarcocysts) in the tissues, primarily the muscles, of intermediate hosts. The definitive host becomes infected through the ingestion of tissues containing mature sarcocysts, and during a sexual phase oocysts or sporocysts are produced in the intestine and shed with feces. Intermediate hosts acquire the infection by ingesting water or food contaminated with sporocysts [2]. In the case of ruminant sarcocystosis, infection can range from mild to severe, resulting in mortality, miscarriage and weight loss and even meat condemnation, depending on a variety of factors, such as parasite species, number of ingested sporocysts, host immunity and the host’s physiological status [1].

Three species belonging to the genus *Sarcocystis* are found in domestic goats: *S*. *capracanis*, *S*. *hircicanis* and *S*. *moulei*. The definitive hosts of the first of these two species are representatives of the Canidae family, while *S*. *moulei* is spread by felids [2]. It has been generally accepted that *Sarcocystis* species infecting domestic sheep (*S*. *arieticanis*, *S*. *gigantea*, *S*. *medusiformis* and *S*. *tenella*) cannot parasitise goats and vice versa [2]. However, recent molecular research implies that some of these *Sarcocystis* species might not be as strictly host-specific as previously assumed [3], suggesting that *Sarcocystis* spp. specific for domestic sheep and goats can be found in wild species of the subfamily Caprinae [4–7].

To our knowledge, Couturier [8] was the first to describe the presence of sarcocysts in Alpine ibex (*Capra ibex* L., 1758), based on the observation of sarcocysts in the myocardium of ibex from the Gran Paradiso National Park, Northwestern Italian Alps in the late 1950s. Since the 1970s, *Sarcocystis* infection in Alpine ibex has been occasionally mentioned or a subject of research [9–17] but to date there has been only one comprehensive study that examined sarcocysts found in muscle tissue samples of Alpine ibex more thoroughly [18]. In that study, Cornaglia et al. used light microscopy to determine a prevalence of 86% *Sarcocystis* infection in samples collected from 54 Alpine ibex in the Grand Paradiso National Park. These authors also used transmission electron microscopy (TEM) to study the morphology of the cyst wall, detecting three types of sarcocysts, corresponding to *S*. *capracanis*, *S*. *cornagliai* and, putatively, *S*. *arieticanis*, in 70%, 27% and 3% of the samples, respectively [18]. Given that various *Sarcocystis* species have similar morphological characteristics, morphological analyses alone are insufficient to identify and distinguish the species of the genus *Sarcocystis* in the subfamily Caprinae; rather, microscopy and molecular methods need to be combined to differentiate these parasites.

Intensive hunting and poaching in the past has almost brought the Alpine ibex to extinction. At the beginning of the 19th century, there were no more than 100 individuals left in an area in the western part of the Alps. However, active protection measures, first introduced in 1911, have formed the basis of the restoration of the species in the entire Alpine Mountain range [19, 20]. Nowadays, Alpine ibex occur again across the entire Alpine arc, from France in the west to Austria in the east, with a total population of about 52,000 individuals [20]. Despite this successful restoration of the species following the bottleneck, concerns about the susceptibility of Alpine ibex to disease have arisen in recent years, as disease outbreaks, such as sarcoptic mange in the Eastern Alps, can cause a severe population decline, with endangered and reintroduced species especially vulnerable [21].

The aim of our study was to perform a detailed morphological and molecular characterisation of *Sarcocystis* species found in the muscles of Alpine ibex in Austria.

## Methods

### Biological material

In the course of a survey of helminth parasitism of Alpine ibex from Austria during the period 2019–2021 [22, 23], we collected muscle tissue (myocardium and diaphragm) samples of ibex from the Pitztal valley located in the central Alps in Tyrol (main location: St. Leonhard im Pitztal; 47°3′58.802″N, 10°50′50.246″E) following regular harvest during the hunting season. The ibex population of the Pitztal was founded with individuals translocated from the Pontresina area in Switzerland in the 1950s, as described elsewhere [22, 24]. The tissue samples from eight ibex were examined for sarcocysts. Until microscopical analysis, the tissue samples were stored frozen (− 20 °C). Specimens were shipped frozen to the Nature Research Centre in Lithuania where detailed morphological and molecular investigations were carried out in 2021–2024.

### Morphological characterisation of sarcocysts from Alpine ibex

Ten sarcocysts were removed from freshly squashed preparations of the myocardium and diaphragm tissues of seven animals using fine needles under the light microscope. The excised sarcocysts were morphologically characterised in fresh preparations by light microscopy based on the shape and size of the cysts and the bradyzoites that emerged from the cysts, as well as on the structure of the cyst wall. The isolated sarcocysts were transferred to individual 1.5-ml vials containing 96% ethyl alcohol until DNA extraction. Sarcocyst morphology and structures were further examined using conventional histology and TEM. For TEM, three additional sarcocysts showing different morphological types were fixed in 2% glutaraldehyde, subsequently postfixed in 1% osmium tetroxide, dried and infiltrated with epoxy resin. Sections were cut using a Leica UC6 ultramicrotome (Leica Microsystems GmbH, Wetzlar, Germany) and stained with 4% uranyl acetate and 3% lead citrate solution. Grids were imaged at 120 kV with the Talos L120C TEM (Thermo Fisher Scientific, Waltham, MA, USA). Prior to histological examination, 1 μm-thick sections were stained with 1% alkaline toluidine blue O solution. Histological and TEM procedures were performed at the National Centre of Pathology (Vilnius, Lithuania).

### Molecular characterisation of sarcocysts

Genomic DNA was extracted from 10 individual sarcocysts using the GeneJET Genomic DNA Purification Kit (Thermo Fisher Scientific Baltics, Vilnius, Lithuania) according to the manufacturer’s tissue protocol. All isolates were subjected to PCR amplification of the partial 18S ribosomal RNA (rRNA) gene and cytochrome* c* oxidase subunit I (*cox1*) sequences. Partial 18S rRNA gene sequences were amplified with the SUNIF1/SUNIR1 and SUNIF3/SUNIR2 primer pairs [25]. The resulting sequences were combined into a single fragment of the 18S rRNA gene. Partial *cox1* sequences were amplified using the SF1 forward primer combined with one of the following reverse primers: SR8D, SR11 and SsunR3 [26–28]. PCR reactions were carried out with Rapid Taq Master Mix (Vazyme, Nanjing, China) according to the manufacturer’s instructions and specified PCR cycling conditions. The evaluation of PCR results, purification of amplified products and sequencing was conducted as described previously [29]. All sequences generated in the present study are available in GenBank with accession numbers PQ963161–PQ963170 (18S rRNA gene) and PQ998228–PQ998236 (*cox1*).

### Phylogenetic analyses

The sequences obtained were compared with those of *Sarcocystis* species using the online Nucleotide BLAST program (http://blast.ncbi.nlm.nih.gov/). For the phylogenetic analyses, the sequences were compared with homologous sequences of numerous *Sarcocystis* species. The number of sequences from the same species was reduced to generate a smaller dataset to allow for more accurate phylogenetic analysis. Multiple sequence alignments were generated with the MUSCLE algorithm available in MEGA11 software [30]. The best-fitting DNA evolution models were selected, and phylogenetic trees were constructed using the maximum likelihood phylogeny inferred by IQ-TREE with ultrafast bootstrap approximation [31–33].

## Results

### *Sarcocystis* species isolated from Alpine ibex

Microscopic sarcocysts were detected in samples from seven of the eight (87.5%) examined animals. Eight sarcocysts were isolated from the diaphragm and two from the myocardium. Sarcocysts from myocardial tissue were found to be short and wide, and those from diaphragm tissue were long and narrow. Three morphological types of sarcocysts were discerned by light microscopy. Sarcocysts attributed to types I and II had finger-like cyst-wall protrusions, while type III sarcocysts had hair-like cyst-wall protrusions. Further investigations revealed that type I sarcocysts belonged to *S*. *capracanis*. Sequences of the 18S rRNA gene and *cox1* of type II sarcocysts significantly differed from all other sequences of *Sarcocystis* spp. available in NCBI GenBank database. However, type II sarcocysts corresponded morphologically to those of *S*. *cornagliai*, which were first identified in Alpine ibex and Alpine chamois (*Rupicapra rupicapra*) [18, 34]. Further TEM and molecular analysis revealed that the type III sarcocysts belong to an unknown species which, during this study, was described for the first time and named as *Sarcocystis rossii* n. sp. Two of the species detected, *S. capracanis* and *S*. *rossii* n. sp., were found in both the diaphragm and the myocardium muscles, whereas *S*. *cornagliai* was found only in the diaphragm of one animal.

### Morphological description of* Sarcocystis capracanis*

**Fig. 1:**
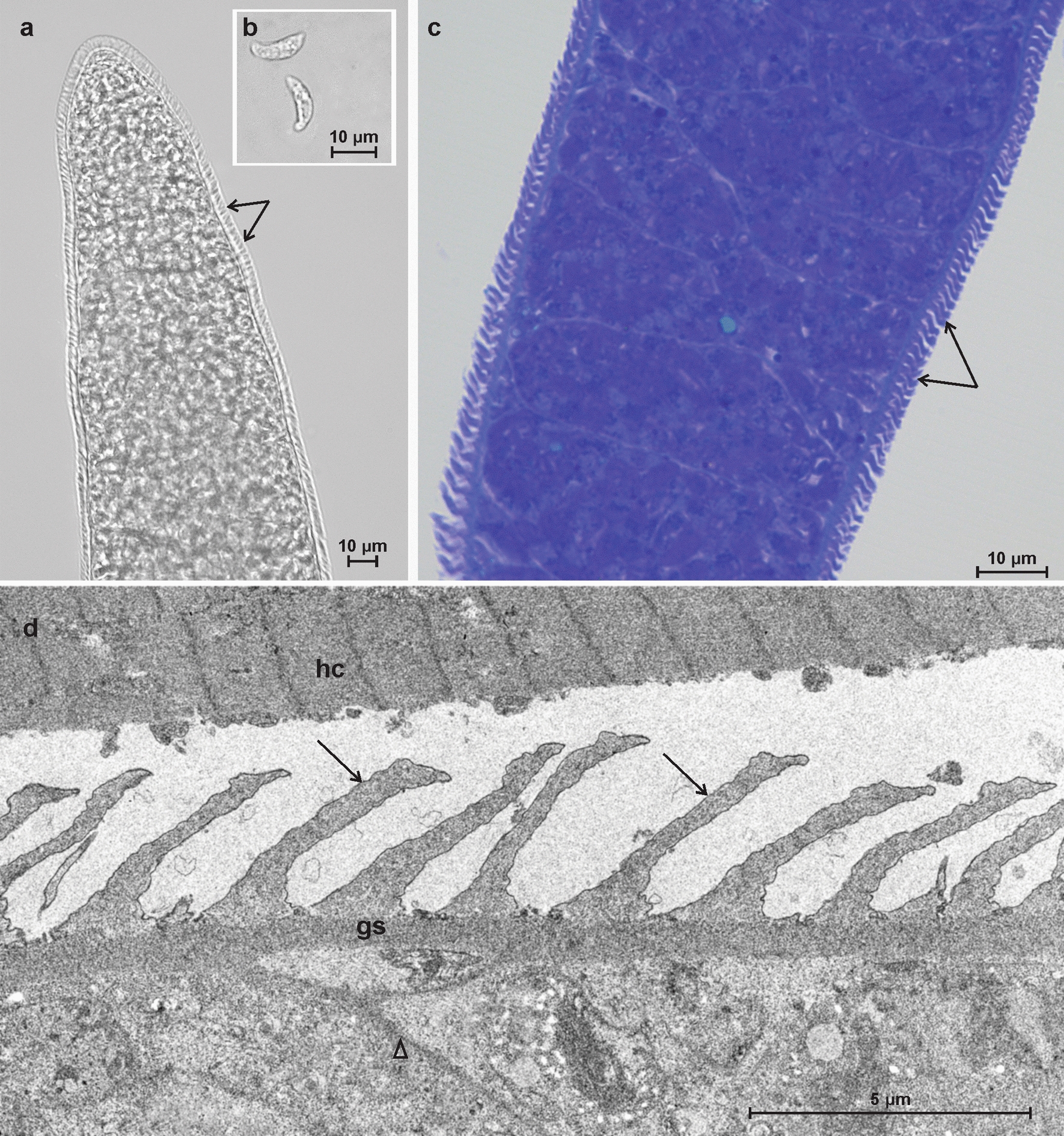
Morphological features of a sarcocyst of *Sarcocystis capracanis* found in diaphragm of Alpine ibex (*Capra ibex*) from Austria. **a**, **b** Light microscopy analysis of a fresh muscle preparation showing a sarcocyst fragment with finger-like cyst-wall protrusions (arrows) (**a**) and banana-shaped bradyzoites (**b**). **c** Toluidine blue staining. Sarcocyst fragment with finger-like protrusions (arrows). **d** Transmission electron microscopy micrograph showing a cyst wall fragment with several finger-like villar protrusions (arrows). Note septa (arrowhead), muscular host cell (*hc*) and ground substance (*gs*)

Sarcocysts of *S*. *capracanis* conformed to the morphology known for this species. The sarcocysts were cigar-shaped with blunt tips and measured 740 × 65 µm (range: 300–1600 × 40–100 µm; *n* = 6). Finger-like protrusions about 4-µm-long were present on the the cyst wall (Fig. 1a). Sarcocysts had clearly visible septa, with groupings of numerous banana-shaped bradyzoites (12.6 × 4.1 μm; range: 10.0–14.7 × 3.3–5.4 μm; *n* = 36) (Fig. 1b). Examination of toluidine blue-stained tissue sections revealed that the sarcocyst was thick-walled due to the presence of upright finger-like protrusions (Fig. 1c). TEM analysis revealed that the villar protrusions (vp) of the sarcocysts wall were upright and finger-like (Fig. 1d). The vp were densely packed, and measured from 3.7 to 5.2 μm in length and from 0.7 to 1.9 μm in width, appearing longer and narrower in some areas and wider and shorter in others. The ground substance layer measured 0.4–0.7 μm in thickness. The sarcocyst wall corresponds to type 14 of the Dubey et al. [2] classification.

### Molecular diagnosis of *Sarcocystis capracanis*

The four 18S rRNA gene sequences of *S*. *capracanis* obtained from Alpine ibex shared 99.7–99.9% identity between themselves and had 97.5–100% identity with other sequences of this species available in GenBank (Table 1). The four resulting *cox1* sequences shared 98.1–99.4% identity and demonstrated 97.2–100% identity compared to other isolates of *S*. *capracanis*. When comparing our *S*. *capracanis* sequences with those of the closely related *S. tenella*, 96.5–98.7% and 91.0–93.9% similarity was established in the 18S rRNA gene and *cox1*, respectively.

**Table 1:** Genetic characterisation of *Sarcocystis* spp. from the Austrian Alpine ibex (*Capra ibex*)

*Sarcocystis* species	Gene	GenBank accession number	Length (bp)^a^	Intraspecific similarity^b^	Similarity to closest *Sarcocystis* species
*S. capracanis*	18S rRNA gene	PQ963167–PQ963170	1837	99.7–100% (97.5–100%)	96.5–98.7% with *S*. *tenella* (from Caprinae)
	*cox1*	PQ998233–PQ998236	1029^c^	98.1–99.4% (97.2–100%)	91.0–93.9% with *S*. *tenella* (from Caprinae)
*S. cornagliai*	18S rRNA gene	PQ963165–PQ963166	1855	100%	93.5–93.6% with *S*. *hardangeri* (from Cervidae)
	*cox1*	PQ998232	1077^d^	–	76.1–77.1% with *S*. *dehongensis* (from Bovidae) 76.2% with *S*. *mihoensis*–like (from domestic sheep) 73.8–75.5% *S*. *ovalis* (from Cervidae) 75.3% with *S*. *frondea* (from Cervidae)
*S. rossii* n. sp.	18S rRNA gene	PQ963161–PQ963164	1832	100%	96.4–97.4% with *S*. *arieticanis* (from Caprinae)
	*cox1*	PQ998228–PQ998231	894^e^ 1029^c^	98.8–99.7%	80.1–88.3% with *S*. *arieticanis* (from Caprinae) 86.2–88.0% with *S*. *hircicanis* (from domestic goat)

*cox1* Cytochrome* c* oxidase subunit I gene,* rRNA* ribosomal RNA

^a^The reverse primer used for *cox1* amplification

^b^The first range of values show the comparison between sequences of the same species determined in the present study; the values in parentheses show the comparison with available sequences of the same species from GenBank

^c^SR8D primer

^d^SR11 primer

^e^SsunR3 primer

### Morphological description of* S. cornagliai*

**Fig. 2:**
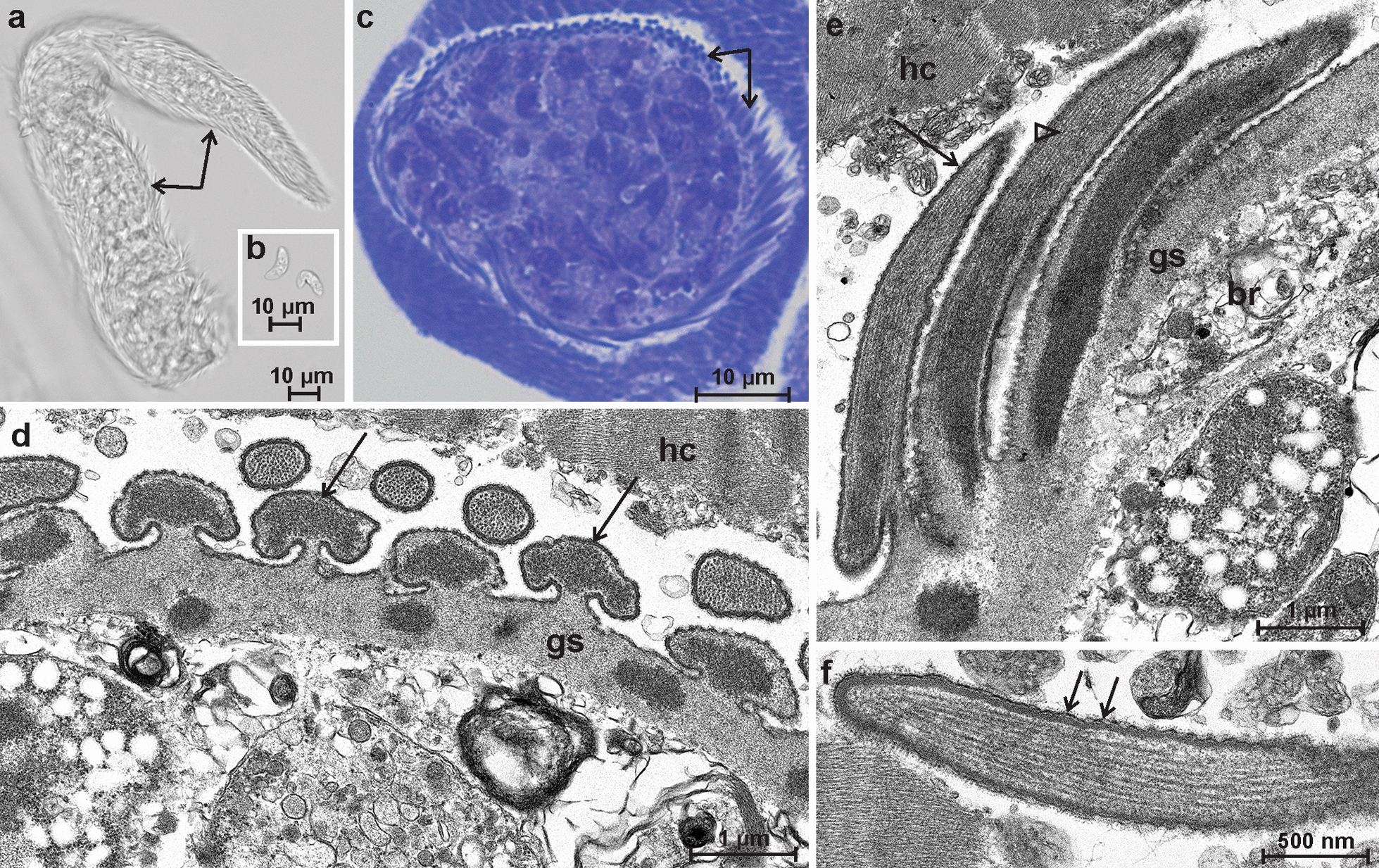
Morphological features of a sarcocyst of *Sarcocystis cornagliai* found in the diaphragm of Alpine ibex (*Capra ibex*) from Austria. **a**, **b** Light microscopy analysis of a fresh muscle preparation showing a sarcocyst fragment with finger-like cyst wall protrusions (arrows) (**a**) and banana-shaped bradyzoites (**b**). **c** Toluidine blue staining. Sarcocyst, showing both cross and longitudinal sections of finger-like protrusions (arrows). **d**, **e**, **f** Transmission electron microscopy micrographs. **d** Cross section of a cyst-wall fragment with mushroom-like villar protrusions (arrows). **e** Longitudinal section of the finger-like protrusions (arrow), which contain an abundance of microtubules in their core (arrowhead); note muscular host cell (*hc*), bradyzoite (*br*) and ground substance (*gs*). **f** A view of a protrusion fragment. The parasitophorous vacuolar membrane contains small knob-like blebs (arrows)

Sarcocysts of *S*. *cornagliai* were spindle-shaped with pointed tips. The sarcocysts of this species were the smallest ones detected from Alpine ibex, measuring 300 × 40 µm (range: 190–400 × 35–45 µm; *n* = 4) and presenting finger- or palisade-like protrusions that were 4- to 6-µm long (Fig. 2a). Sarcocysts had clearly visible septa, which divided sarcocysts into chambers containing banana-shaped (10.2 × 3.6 μm; range: 7.8–12.7 × 3.4–4.1 μm; *n* = 14) bradyzoites (Fig. 2b). In toluidine blue-stained tissue sections, the sarcocyst was thick-walled with mushroom- or finger-like vp (Fig. 2c). By TEM, in cross-section, the sarcocyst wall was covered with mushroom-like-shaped vp with a dense heap/core of microtubules in the central area (Fig. 2d). In places where bundles of microtubules were severed, electron-dense circular areas were seen within the ground substance. In the longitudinal sections, the protrusions were about 5.5–6 μm long and 0.6–0.7 μm wide at their base, with gradual narrowing towards the tips (Fig. 2e). The protrusions were bent and folding over the cyst wall. Microtubules extended into the ground substance from the sarcocyst wall protrusions. The surface of the protrusions seemed to be serrated since the parasitophorous vacuolar membrane had small knob-like blebs (Fig. 2f). There were gaps of approximately 0.9–1.3 μm between the protrusions of the sarcocyst wall. The ground substance layer measured approximately 0.5–0.9 μm. The sarcocyst wall was identified as type 24 of the Dubey et al. [2] classification.

### Molecular characterisation of *Sarcocystis cornagliai*

The two 18S rRNA gene sequences of *S*. *cornagliai* obtained in the present study were 100% identical (Table 1). These sequences differed considerably from the sequences of other known *Sarcocystis* species. Comparison of 18S rRNA gene sequences showed that the closest sequence similarity of these two gene sequences was to sequences of *S*. *hardangeri* (93.5–93.6%). We were able to amplify only one *cox1* sequence of *S*. *cornagliai*, which had the highest similarity with *S*. *dehongensis* (76.1–77.1%), followed by 76.2% similarity to *S*. *mihoensis*-like (MK420016), 73.8–75.5% similarity to *S*. *ovalis* and 75.3% similarity to *S*. *frondea* (Table 1).

### Morphological description of *Sarcocystis rossii* n. sp.

**Fig. 3:**
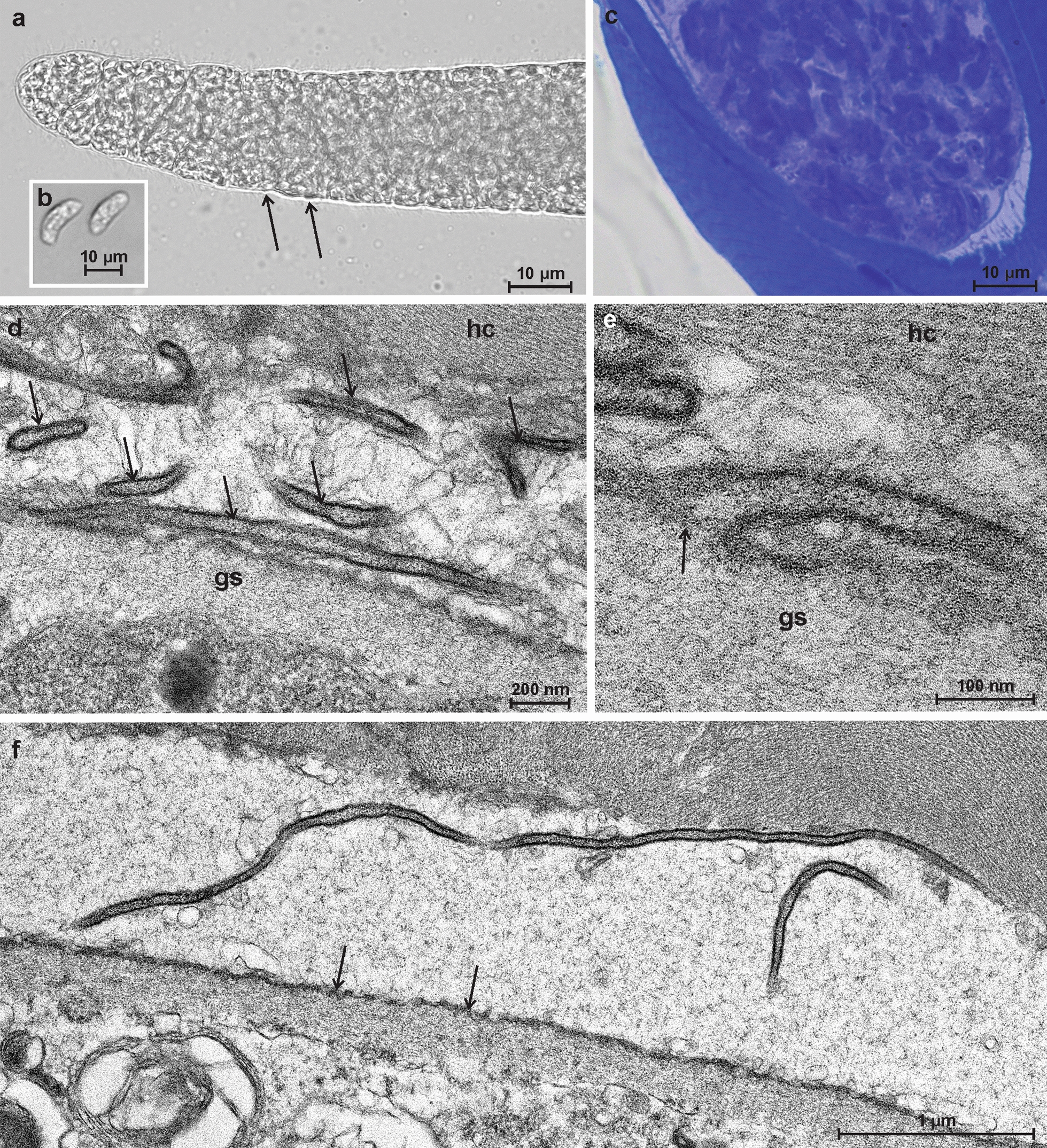
Morphological features of a sarcocyst of *Sarcocystis rossii* n. sp. found in the diaphragm of Alpine ibex (*Capra ibex*) from Austria. **a**, **b** Light microscopy analysis of a fresh muscle preparation showing a sarcocyst fragment with hair-like cyst-wall protrusions (arrows) (**a**) and banana-shaped bradyzoites (**b**). **c** Toluidine blue staining. A fragment of the thin-walled sarcocyst was filled with bradyzoites. **d**, **e**, **f** Transmission electronic microscopy micrographs. **d** A cyst-wall fragment with intermittent hair-like cyst-wall villar protrusions (*vp*) folded over the cyst wall (arrows). **e** An enlarged view of the hair-like vp near the base of the sarcocyst wall. Vp were determined to be filled with numerous electrondense granules; note cone-shaped widening (arrow). **f** A view of a cyst-wall fragment and the detached long hair-like vp; note small blebs of the parasitophorous vacuolar membrane (arrows), muscular host cell (*hc*) and ground substance (*gs*)

Sarcocysts of *S. rossii* n. sp. measured 1130 × 68 µm (range: 350–2200 × 35–100 µm; *n* = 7) and were ribbon-shaped with pointed tips. Examination of the freshly squashed preparations revealed that hair-like protrusions about 5 µm long were present on the sacrocyst wall (Fig. 3a). Sarcocysts occasionally appeared to have a smooth wall, as hair-like protrusions were easily broken off during cyst preparation for microscopy examination. Sarcocysts also had clearly visible septa, which divided sarcocysts into chambers containing banana-shaped (12.5 × 4.5 μm; range: 9.4–13.9 × 3.4–5.2 μm; *n *= 12) bradyzoites (Fig. 3b). In toluidine blue-stained tissue sections, the sarcocyst of *S. rossii* n. sp. was thin-walled (Fig. 3c). TEM revealed the presence of hair-like vp on the cyst wall of a *S. rossii* n. sp. sarcocyst (Fig. 3d); the protrusions were 50 nm wide, and their cores were filled with many fine electron-dense granules. The vp arose from the dome-shaped bases, the width of which was about 100 nm (Fig. 3e). The length of the protrusions was difficult to measure due to their discontinuity; the largest recorded protrusion was 4.9 μm (Fig. 3f). The parasitophorous vacuolar membrane of the cyst wall had tiny blebs. The ground substance layer was very thin, measuring 0.2–0.4 μm and extending into the cyst as septa. The cyst wall of this sarcocyst was attributed to type 7a of the Dubey et al. [2] classification.

### Molecular characterisation of *S*. *rossii* n. sp

The four 18S rRNA gene sequences of *S. rossii* n. sp. were 100% identical (Table 1). Compared to other nearly complete sequences of 18S rRNA gene available in GenBank, the sequences of *S. rossii* n. sp. were most similar to those of numerous *Sarcocystis* species (*S*. *arieticanis*, *S*. *hircicanis*, *S*. *cruzi*, *S*. *poephagicanis*, *S*. *capracanis*, *S*. *gjerdei*, *S*. *levinei*, *S*. *tenella*) having ungulates as intermediate hosts, reaching up to 97.4% similarity. The highest sequence similarity of *S. rossii* n. sp. within the 18S rRNA gene was established when compared to nearly complete *S*. *arieticanis* sequences (96.4–97.4%). The *cox1* sequences of *S. rossii* n. sp. shared 98.8–99.7% identity between themselves and had the highest similarity to sequences of *S*. *arieticanis* and *S*. *hircicanis*, with a range of 80.1% to 88.3% and 86.2% to 88.0%, respectively.

Sarcocysts morphologically similar to those of *S*. *rossii* n. sp. have previously been found in Alpine ibex (Figs. 5 and 6 in [18]); however, at that time they were thought to belong to *S*. *arieticanis*. Comparing *S. rossii* n. sp. to *S*. *arieticanis*, we noted significant genetic differences in both the 18S rRNA gene (≥ 2.6%) and *cox1* (≥ 11.7%). Taking into account sarcocyst morphology and genetic data, we therefore propose a new *Sarcocystis* species, *S. rossii* for an organism observed in the muscles of Alpine ibex in Austria.

**Taxonomic summary** of *Sarcocystis rossii* n. sp.

**Type intermediate host** Alpine ibex (*Capra ibex* L., 1758).

**Definitive host** Unknown, based on phylogeny probably canids.

**Locality** Pitztal valley (Northern Tyrol), Austria.

**Type specimen** Hapantotype 1 slide toluidine blue-stained (NRCP00003) is deposited in the Nature Research Centre, Vilnius, Lithuania.

**Sequences** deposited in NCBI GenBank with accession numbers PQ963161–PQ963164 (18S rRNA gene) and PQ998228–PQ998231 (*cox1*).

**Etymology** The species was named in honor of Dr. Luca Rossi, who had a long career studying wild mountain ungulates and made significant contributions to the knowledge of their parasites.

**ZooBank registration** The Life Science Identifier (LSID) of the article is urn:lsid:zoobank.org:pub:2735883E-FC58-49E5-A586-0E36F96BBE7C. The LSID for the new name *Sarcocystis rossii* is urn:lsid:zoobank.org:act:7A0DF4DC-5CDF-450C-9647-EEDE91E82C69.

### Phylogeny

**Fig. 4:**
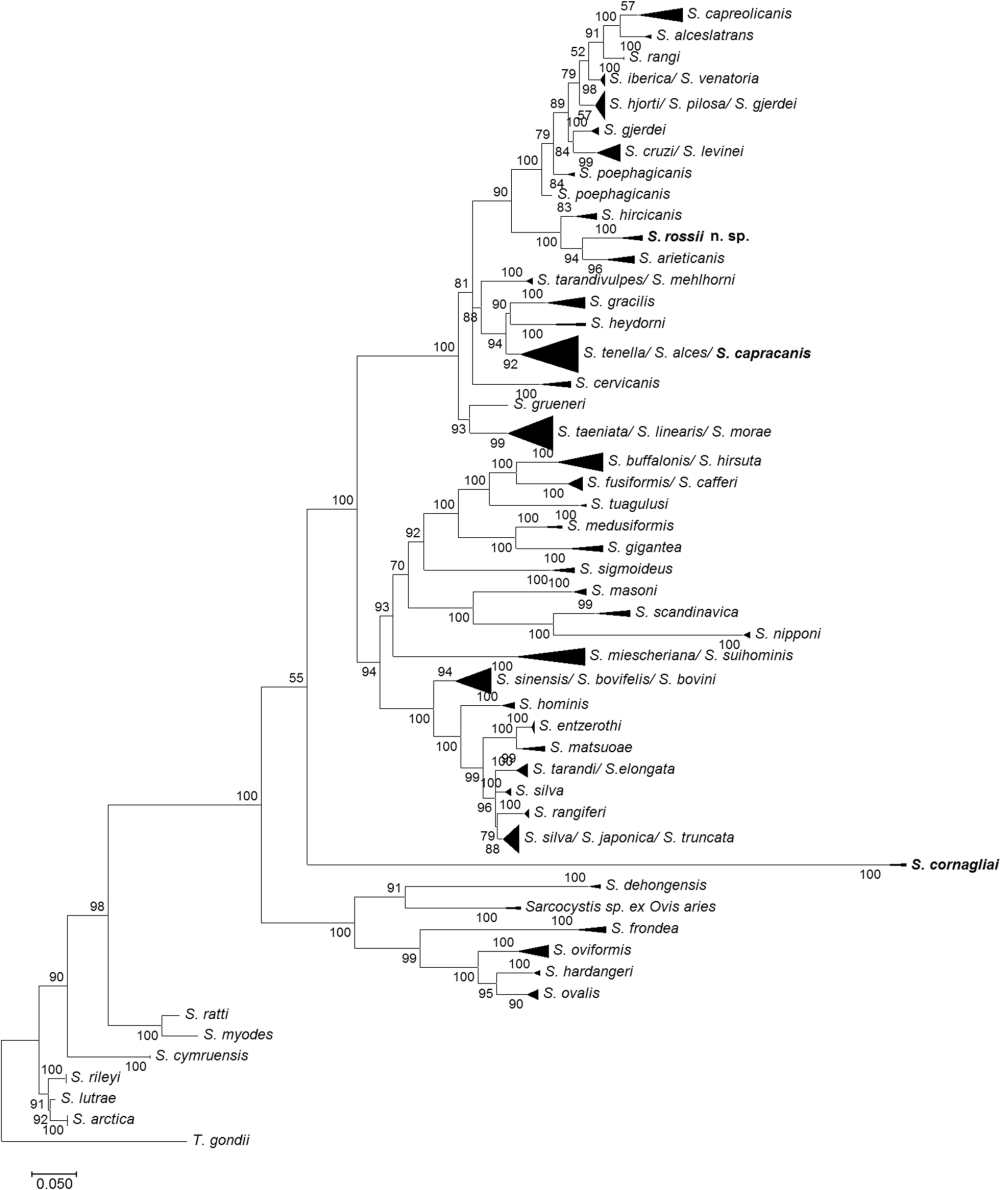
Phylogenetic tree of selected *Sarcocystis* species based on 18S ribosomal RNA gene sequences. The tree was constructed using the maximum likelihood method, rooted on *Toxoplasma* *gondii*. The HKY + F + I + G4 evolutionary model was set for the phylogenetic analysis. The bootstrap values are indicated next to branches. *Sarcocystis* spp. obtained in this study are shown in bold font

**Fig. 5:**
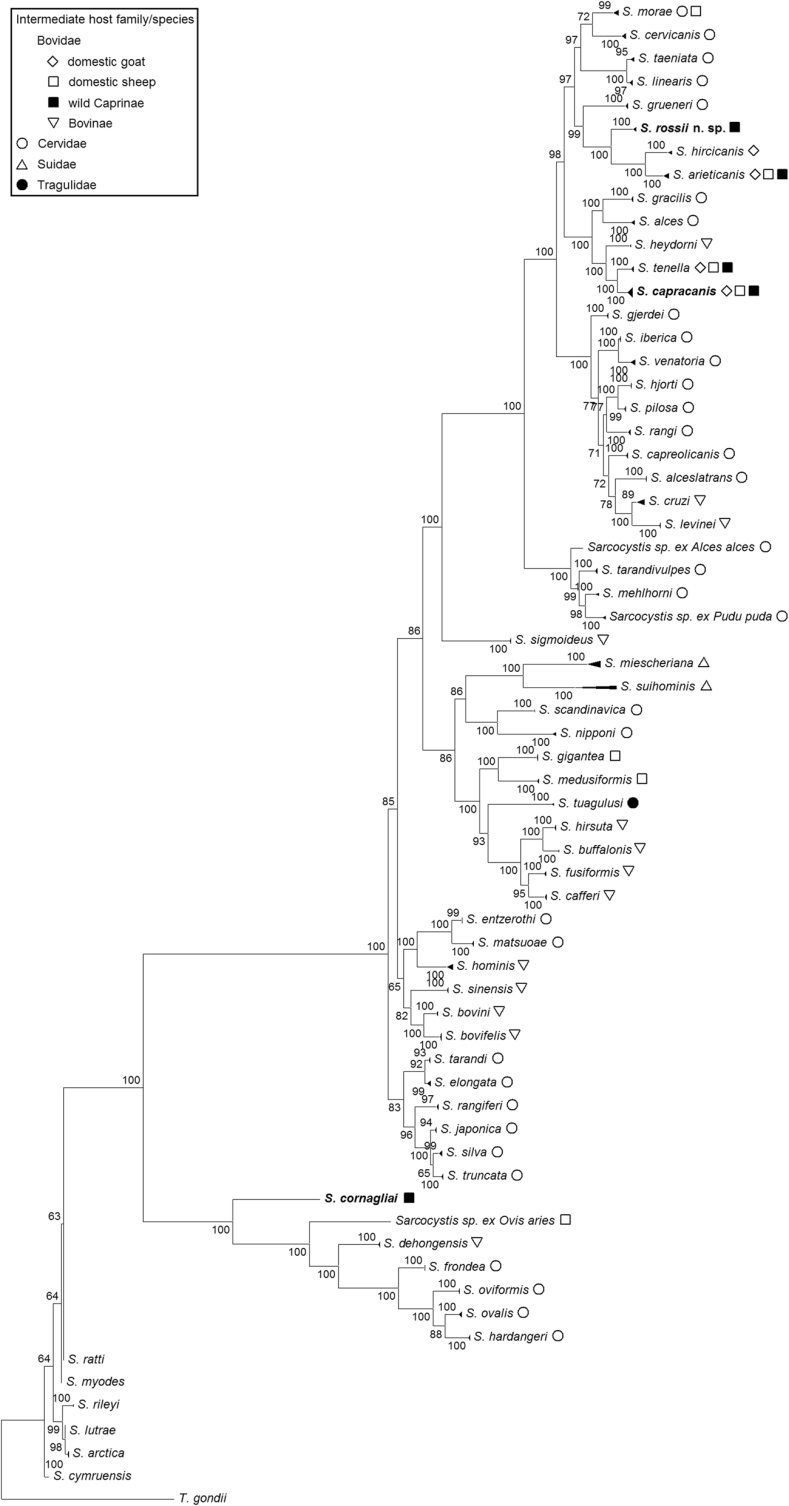
Phylogenetic tree of selected *Sarcocystis* species based on cytochrome c oxidase I gene (*cox1*) sequences. The tree was constructed using the maximum likelihood method, rooted on *T*. *gondii*. The K2P + I + G4 evolutionary model was set for the phylogenetic analysis. The bootstrap values are indicated next to branches. *Sarcocystis* spp. obtained in this study are shown in bold font. The intermediate host in which the *Sarcocystis* species was found is indicated by a geometric shape after the species name

Phylogenetic trees were built using both molecular loci, the 18S rRNA gene and *cox1* (Figs. 4 and 5). The final 18S rRNA gene sequence alignment included 378 sequences and 1930 aligned nucleotide positions. The phylogenetic analysis based on *cox1* sequences contained 274 sequences and 790 aligned nucleotide positions. The sequences used for the phylogenetic analyses are given in supplementary material (Additional file 1: Table S1). In contrast to *cox1*, phylogenetic relationships of some *Sarcocystis* species were not fully defined using 18S rRNA gene sequences (Figs. 4 and 5). The 18S rRNA gene sequences were not sufficiently variable to discriminate *S*. *capracanis* from *S*. *alces* and *S*. *tenella* in the phylogenetic analysis (Fig. 4). Moose (*Alces alces*) and domestic sheep are proven intermediate hosts of *S*. *alces* and *S*. *tenella*, respectively [2, 35]. On the contrary, *cox1* sequences of *S*. *capracanis* from Alpine ibex clustered with other sequences of this species and was found to be a sister taxon to *S*. *tenella*. *Sarcocystis cornagliai* has established a separate branch in both phylograms. Based on 18S rRNA gene analysis, the phylogenetic placement of *S*. *cornagliai* was not supported by a significant bootstrap value. In the *cox1* phylogenetic tree, *S*. *cornagliai* clustered with *S*. *dehongensis* from water buffalo (*Bubalus bubalis*), with *S*. *mihoensis*-like from domestic sheep and with several *Sarcocystis* species from various cervids, i.e. *S*. *frondea*, *S*. *hardangeri*, *S*. *ovalis* and *S*. *oviformis*. These findings show that *S*. *cornagliai* is closely related to *Sarcocystis* species that are transmitted by corvid birds. Based on both phylogenetic trees, *S*. *rossii* n. sp. formed a separate branch and was most closely related to *S*. *arieticanis* and *S*. *hircicanis* from sheep and goat, respectively. At the 18S rRNA gene, sequences of *S*. *rossii* n. sp. grouped with those of *S*. *arieticanis*, while at *cox1, S*. *rossii* n. sp. formed a sister branch to a clade consisting of *S*. *arieticanis* and *S*. *hircicanis*. In general, *S*. *rossii* n. sp. shared close relationships with species that use canids as definitive hosts.

## Discussion

Using light microscopy, TEM, molecular analyses of the 18S rRNA gene and *cox1*, we identified *S*. *capracanis*, *S*. *cornagliai* and the novel species *S*. *rossii* n. sp. in muscle tissues of Alpine ibex. This study also provides the first molecular characterisation of *S*. *cornagliai*, a species that was described three decades ago based solely on morphological parameters.

A comparison of the morphology of the *Sarcocystis* species reported in the present study work to those described by Cornaglia et al. [18] in an earlier study revealed that the three species identified in our study correspond to the three sarcocyst types reported by Cornaglia et al. [18]: *S*. *cornagliai* to type I, *S*. *capracanis* to type II and *S*. *rossii* n. sp. to type III. At that time, Cornaglia et al. [18] considered that the sarcocysts of the latter type might have been those of *S*. *arieticanis*. Most *Sarcocystis* species having sarcocysts with hair-like protrusions are practically indistinguishable in terms of morphological characteristics by light microscopy and TEM, but molecular analysis has enabled various morphologically identical *Sarcocystis* species to be distinguished [36, 37]. In the present study, *S*. *rossii* n. sp. sequences differed significantly from those of other *Sarcocystis* species, particularly in the *cox1* region.

Novel genes for differentiating *Sarcocystis* species are being sought continually [38]. There is currently no single gene that allows all species of the genus *Sarcocystis* to be distinguished. The intermediate host of *Sarcocystis* spp. determines the genetic region used to identify the parasite. The 18S rRNA gene is historically the most often used gene for characterisation of *Sarcocystis* species, but it is insufficient to distinguish closely related *Sarcocystis* species [39]. The 28S rRNA gene is often used to identify *Sarcocystis* species having rodents as intermediate hosts [25]. The internal transcribed spacer 1 (ITS1) region is most effective at distinguishing *Sarcocystis* spp. from predatory mammals and birds [40, 41], whereas mitochondrial *cox1* is effective for identifying and uncovering the phylogenetic relationships of *Sarcocystis* parasites in ruminants [26, 42]. Several other genes have been proposed to characterise *Sarcocystis* using nuclear, mitochondrial and apicoplast DNA loci [25, 38], but so far only a few species have been tested with these genes, and the effectiveness of novel target genes warrants additional investigation. In the present study, *S*. *cornagliai* showed considerable genetic differences from other *Sarcocystis* species within the 18S rRNA gene and *cox1*. The latter gene also helped to clearly distinguish *S*. *rossii* n. sp. and *S*. *capracanis* from other *Sarcocystis* species, but only minor genetic differences (< 3%) were detected within the 18S rRNA gene when isolates from the most closely related species were compared. Furthermore, *cox1* has been found to be better suited for phylogenetic analysis, whereas the 18S rRNA gene analysis failed to distinguish phylogenetic relationships of numerous *Sarcocystis* species, as previously demonstrated [43].

*Sarcocystis cornagliai* was first described using light microscopy and TEM, and its ultrastructure led to its classification as type 24 of Dubey et al. [2] [34]. The typical appearance of type 24 is characterised by a mushroom-like vp of the cyst wall with a core of tightly packed microtubules. However, this can only be seen in cross sections of the sarcocyst wall. In longitudinal sections, we believe *S*. *cornagliai* is similar type 11c of Dubey et al. [2], which has been suggested for sarcocysts of *S*. *bertrami*. Similarly, in cross-section, the sarcocyst wall of *S*. *frondea* (type 39 of Dubey et al. [2]) appears to have mushroom-like protrusions; yet, in longitudinal sections, the cyst wall of this species presents leaf-like protrusions [44]. The molecular characterisation of *S*. *cornagliai* in this study shed light on the taxonomy of morphologically similar *S*. *mihoensis*-like species described in sheep. The status of *S*. *mihoensis*-like was unclear due to the possibility of sheep serving as an intermediate host for *S*. *cornagliai* [45]. The molecular data on *S*. *cornagliai* obtained herein demonstrated clear genetic differences from *S*. *mihoensis*-like and other known *Sarcocystis* species. Therefore *S*. *mihoensis* from sheep should be considered as a valid species.

*Sarcocystis* species differ in their specificity for intermediate hosts, complicating the epidemiology of the genus. *Sarcocystis capracanis* is typically associated with domestic goats but has also been found in domestic sheep [3] and wild species of the Caprinae subfamily, including Barbary sheep (*Ammotragus lervia*) and European mouflon (*Ovis gmelini musimon*) [6, 7]. The present study set a new host record for *S*. *capracanis*. This *Sarcocystis* species is known to be transmitted through canids [2]. There is only one relevant wild canid in the Austrian Alps of Northern Tyrol with widespread occurrence, namely the red fox (*Vulpes vulpes*). In addition, there have been several records of gray wolves (*Canis lupus*) and few sightings of golden jackal (*Canis aureus*); however, the two species did not establish in the region [46].

In addition to the Alpine ibex, *S*. *cornagliai* has previously been detected in esophagus and diaphragm tissue of the Alpine chamois [34], and sarcocysts morphologically resembling *S*. *cornagliai* have been described in tongue and skeletal muscle samples of the mountain goat (*Oreamnos americanus*) in the USA [47, 48]. Although molecular studies on *Sarcocystis* from other caprine would be required to confirm this latter finding, it would appear that *S*. *cornagliai* may use several wild hosts of the Caprinae subfamily. A large-scale experimental study was conducted in which mountain goat tongue tissue containing sarcocysts resembling *S*. *cornagliai* were fed to several species of canids, felids, ursids, procyonids, mustelids, Old World monkeys and birds of prey, but the definitive host for this *Sarcocystis* species remained unidentified [47]. The phylogenetic analysis conducted in the present study (Fig. 5) revealed that *S*. *cornagliai* sequences cluster with those of *S*. *dehongensis* from water buffalo (*Bubalus bubalis*), *S*. *mihoensis*-like from domestic sheep and several *Sarcocystis* species from cervids, namely *S*. *frondea*, *S*. *hardangeri*, *S*. *ovalis* and *S*. *oviformis*. Two of these *Sarcocystis* species are known to use corvid birds as their definitive hosts. *Sarcocystis ovalis* was found in magpies (*Pica pica*), jungle crows (*Corvus macrorhynchos*) and hooded crows (*Corvus cornix*), while *S*. *oviformis* was detected in hooded crows and common ravens (*Corvus corax*) [49–51]. Thus, the present study demonstrates that *S*. *cornagliai* is related to *Sarcocystis* species transmitted by corvid birds. In general, *Sarcocystis* species forming sarcocysts in muscle tissues of ungulates and transmitted by birds occur less frequently than those transmitted by canids or felids [45].

In the present study, we described *S*. *rossii* n. sp., whose sarcocysts were characterised as being ribbon-shaped with a presence of hair-like protrusions. Morphologically similar sarcocysts were found in European mouflon [52], but molecular analysis revealed the latter to belong to *S*. *arieticanis* [7]. Further molecular studies in wild Caprinae species are needed to determine whether *S*. *rossii* n. sp. is specific to the intermediate host or can be detected in other wild caprids. In our study, phylogenetic analyses based on the 18S rRNA gene and *cox1* demonstrated that *S*. *rossii* n. sp. clustered with *Sarcocystis* species which use members of the Bovidae and Cervidae as intermediate hosts and canids as their definitive hosts. In a previous experiment in which Alpine ibex muscle tissue containing *Sarcocystis* spp. cysts was fed to canids, felids and birds of prey, the results indicated that canids are the definitive hosts [9]. In another study, lambs and goat kids were infected experimentally with sporocysts recovered from dogs fed *Sarcocystis*-infected Alpine ibex meat, and mature sarcocysts were subsequently identified in the myocardium and skeletal striated muscles of all infected animals [14]. In the current investigation, we found two *Sarcocystis* species, *S*. *capracanis* and *S*. *rossii* n. sp., to be transmitted by canids. It is likely that one of these two species or an as yet unknown species may use both domestic goats and sheep as intermediate hosts.

## Conclusions

In the present study three *Sarcocystis* species, *S*. *capracanis*, *S*. *cornagliai* and *S*. *rossii* n. sp. were identified in tissue samples of Alpine ibex from Austria. Sarcocysts of these three species were characterised morphologically (light microscopy and TEM), molecularly (18S rRNA gene and *cox1*) and phylogenetically. In light of the results of earlier investigations and the findings of the present work, research on *Sarcocystis* speciation and epidemiology in Alpine goats and other wild Caprinae species should be continued.

## Supplementary Information

**Additional file 1: Table S1.** Taxa and GenBank accession numbers of sequences used in the phylogenetic analyses.

## Data Availability

No datasets were generated or analysed during the current study.
