# Supplementary material for: Morphological and molecular characterisation of Sarcocystis capracanis, Sarcocystis cornagliai and Sarcocystis rossii n. sp. infecting the Alpine ibex (Capra ibex)
Source: Parasit Vectors. 2025 Mar 10;18:96. doi: 10.1186/s13071-025-06737-8 (PMC11892209; doi:10.1186/s13071-025-06737-8)
Supplement: Supplementary file 1 — Additional file 1: Table S1. Taxa and GenBank accession numbers of sequences used in the phylogenetic analyses. [file 13071_2025_6737_MOESM1_ESM.docx]

**Table S1** Taxa and GenBank accession numbers of sequences used in the phylogenetic analyses. Sequences obtained in the present study are in boldface

| Species/taxon | 18S rRNA gene sequences | *cox1* sequences |
| --- | --- | --- |
| *Sarcocystis alces* | EU282018, KF831273−KF831274 | KC209587, KF831244, MK234149−MK234150 |
| *Sarcocystis alceslatrans* | EU282033, KF831275−KF831276 | KC209588, KC209590−KC209591, KF831248, KF831250 |
| *Sarcocystis arctica* | KF601301, KF601304, KX022100, MF596218, MF596228, MF596231, MZ329343, OR921258 | KF601318, KY947304, MF596289, MZ332967, PQ217791, PQ243234 |
| *Sarcocystis arieticanis* | L24382, MF039330, MH41303−MH413036, MK420017, PQ538540 | MH413047, ON858957, ON858961−ON858962, PQ165949 |
| *Sarcocystis bovifelis* | KC209743−KC209744, KT901125, KT901127, KT901132−KT901133, KT901138 | KT900964, KT900994, KT900998, MT796923, MT796925, OR591723, OR591726 |
| *Sarcocystis bovini* | KT901142, KT901144, KT901147−KT901148, KT901155 | KT901004, KT901012, KT901016, LC171858 |
| *Sarcocystis buffalonis* | AF017121, KU247904, KU247907, KU247909−KU247910, KU247913 | KU247868, MG792802 |
| *Sarcocystis cafferi* | KJ778014−KJ778017, KJ778019 | KJ778020, KJ778022, KJ778025, KJ778027−KJ778028 |
| *Sarcocystis capracanis* | KU820982−KU820983, L76472, MW832479, MW832489, MW832493−MW832494, OP430804, OP430806, OP430808, **PQ963167−PQ963170** | KU820977, MW848330, MW848335, OP470343, PP668141, **PQ998233−PQ998236** |
| *Sarcocystis capreolicanis* | JN226117−JN226119, JN256129, KY019016, KY019021, KY019027, MN334248, MN334251, MN334254, MN334255 | KC209595, KF241311, MN339288, PP935191, PP935192 |
| *Sarcocystis cervicanis* | KY973336, KY973346, KY973349, KY973351, KY973354 | KY973289−KY973291, KY973294−KY973295 |
| *Sarcocystis cornagliai* | **PQ963165− PQ963166** | **PQ998232** |
| *Sarcocystis cruzi* | AF017120, JX679468, KC209739, KT901167, KT901168, KT901172, LC171827, LC171830, OR553289 | KC209600, LC171860, MK962350, MT796945, OR570879 |
| *Sarcocystis cymruensis* | MT372786, PQ277039 | PQ297885 |
| *Sarcocystis dehongensis* | KY711373−KY711375 | KY711376−KY711377, OR591727−OR591729 |
| *Sarcocystis elongata* | GQ251011, GQ251014, GQ251017, GQ251020 | KF241318−KF241319, KF241327, KF241329, OP617447 |
| *Sarcocystis entzerothi* | KX643334, KX643337, MF596181−MF596182, MN334257, MN334260−MN334262, MN443757−MN443758 | MF596201, MN339294, MN450919, MT070639, OP617434 |
| *Sarcocystis frondea* | MF596183−MF596187 | MF596202−MF596206 |
| *Sarcocystis fusiformis* | KR186116−KR186117, KR186119−KR186120, KR186123, U03071 | KR186114, KU247892, KX574313, MH899163 |
| *Sarcocystis gigantea* | KC209733, L24384, MK420020, MT705975, OP550293 | KC209601, KC209604, MK120979, MK420011, MK420012, MT722970 |
| *Sarcocystis gjerdei* | LC349475−LC349477, LC349479, LC481028−LC481031 | LC349938, LC349940, LC481082−LC481083, LC481085 |
| *Sarcocystis gracilis* | FJ196261, JN256131, KF880741, KY019031, MN334263, MN334267, MN334274, MN334281, MN334289 | KC209614, KF898100, MN339302−MN339303, MN339321 |
| *Sarcocystis grueneri* | EF056010 | KC209615, KC209618, KC209621−KC209623 |
| *Sarcocystis hardangeri* | EF056013, EF056014, EF467654, GQ250987 | KC209627−KC209628, KC209630, KC209632−KC209633 |
| *Sarcocystis heydorni* | KX057996−KX057997 | KX057994−KX057995 |
| *Sarcocystis hircicanis* | KU820984−KU820985, OP430816, OP430818, OP430823 | KU820975−KU820976, OP470342 |
| *Sarcocystis hirsuta* | AF017122, JX855283, KC209741, KT901156, KT901160, KT901162, KT901164, KT901166, MT706003 | KT901042, LC171863, MT722974, MT796946, MT796949 |
| *Sarcocystis hjorti* | EU282017, GQ250990, JN256124, JN256128, KF831294−KF831295, KY973332 | MK234162, MT070644, OP617368, OP617399, OP617424 |
| *Sarcocystis hominis* | JX679470−JX679471, KF954731, MT792481, OR526735 | MH021119, MK497841−MK497842, OR543019, OR543022 |
| *Sarcocystis iberica* | KY973314−KY973315, KY973318, KY973320, KY973323 | KY973286, MT070663, OP617436, OP617438, OP617440 |
| *Sarcocystis japonica* | LC349445, LC349460, LC349464, LC481012, LC830206 | LC349964, LC349973, LC349976, LC481065, OP617452 |
| *Sarcocystis levinei* | KU247914, KU247917, KU247919, KU247921, KU247922 | KU247877−KU247878, KU247885, MH255775, MH255778 |
| *Sarcocystis linearis* | KY019040−KY019041, KY019046, KY019054, KY973357−KY973358, KY973368, MN334293, MN334300 | KY973312, MK234165, MN339324, MN339332, OM906801 |
| *Sarcocystis lutrae* | KM657769 | KM657809 |
| *Sarcocystis masoni* | ON533528−ON533531, ON533533 | − |
| *Sarcocystis matsuoae* | LC349471, LC481022−LC481024 | LC349971−LC481074, LC481076 |
| *Sarcocystis medusiformis* | MK420021, MT705985 | MK420014−MK420015, MT722971−MT722972 |
| *Sarcocystis mehlhorni* | KT378042 | KT378044−KT378045 |
| *Sarcocystis miescheriana* | JN256123, JX840464−JX840465, JX840467, MH404232, MK867454, MT066104, MT066106−MT066107, OQ472889 | LC349978, MH404219, MT070619, OQ472076, PP136048, PQ274669 |
| *Sarcocystis morae* | KY973375, MK790236, MK790238, MK790246, MN443746, MN443748, MN443752−MN443753, MN443755 | KY973313, MN450887, MN450905, MN450916, OP617451 |
| *Sarcocystis myodes* | OM523016 | OM486938 |
| *Sarcocystis nipponi* | MF596188−MF596192 | MF596207−MF596209 |
| *Sarcocystis ovalis* | EU282019, EU282034, GQ250988−GQ250989, LC184602, LC481011, MF596193−MF596194 | KC209646, KC209650−KC209651 KF241364, MF596211 |
| *Sarcocystis oviformis* | FJ196262, JN256130, KC209745−KC209746, KF880742, MN334314−MN334318 | KC209661, KF898109, MN339334, PP935183, PP935189 |
| *Sarcocystis pilosa* | KU753891, KU753893−KU753894, KU753896, KU753898, LC349474, LC466178, LC466180, LC466182−LC466183, LC481025−LC481027, LC496069 | KU753910, LC466199, LC496070, MT070676, OP617448 |
| *Sarcocystis poephagicanis* | OR573620−OR573623 | − |
| *Sarcocystis rangi* | EF056011, EF467655 | KC209663, KC209665, KC209666, KC209669−KC209670 |
| *Sarcocystis rangiferi* | EF056015−EF056016, GQ250978−GQ250979, GQ250982, GQ250984 | KC209672−KC209673, KF241386, KF241393, KF241409 |
| *Sarcocystis ratti* | MK425190 | MK430072 |
| *Sarcocystis rileyi* | GU120092, HM185742, KJ396583, LT992317, LT992320−LT992321, OR416136 | KJ396582, KT184389 |
| *Sarcocystis rossii* | **PQ963161−PQ963164** | **PQ998228−PQ998231** |
| *Sarcocystis scandinavica* | EU282020−EU282021, EU282023−EU282024, EU282027 | KC209684, KC209685 |
| *Sarcocystis sigmoideus* | OR526731−OR526734 | OR543013−OR543014, OR543016−OR543017, OR543025 |
| *Sarcocystis silva* | EU282016, JN226122−JN226125, JN256132, KF880743, KX643339, KY019056−KY019057, KY019061, KY019063−KY019064, MN334320−MN334321, MN334326−MN334327 | KF898113, KY019004, MK234166, MN339350, OP617453 |
| *Sarcocystis sinensis* | JX679466, JX679469, KF954728, KF954730, KT901102, KT901106, KT901110, KT901112−KT901113 | KT900956−KT900958, KT900960, KU196749 |
| *Sarcocystis* sp. ex *Alces alces* | MK224430 | MK234169 |
| *Sarcocystis* sp*.* ex *Ovis aries* | MK420022−MK420023 | MK420016 |
| *Sarcocystis* sp. ex *Pudu puda* | − | MT180293−MT180295 |
| *Sarcocystis suihominis* | MH404229, MK867455, OR120386, PQ253008 | MH404228, OR101956, PQ274657 |
| *Sarcocystis taeniata* | KF831277, KF831282, KF831289, KF831291−KF831292, KT626602, KU753886−KU753888, KU753890 | KF831258, KF831267, KF831269, KU753901 |
| *Sarcocystis tarandi* | EF056017−EF056018, GQ250968−GQ250970, GQ250972, GQ250975 | KC209697−KC209698, KF241415, KF241423, KF241438 |
| *Sarcocystis tarandivulpes* | EF056012, EF467656−EF467657 | KC209713, KC209715−KC209716, KC209720, KC209722 |
| *Sarcocystis tenella* | KC209735−KC209737, KP263752, KP263755, KP263759, MF039329, MH413034, MK420018−MK420019, MW832471, MW832473, PQ538521 | KC209731, MK419980, MW848315, MW848318−MW848319 |
| *Sarcocystis truncata* | GQ251022−GQ251023, GQ251027, GQ251030, MF596195−MF596197 | KC209681, MF596214, MF946585, OP617441, OP617444 |
| *Sarcocystis tuagulusi* | KT893710−KT893711 | KT893708−KT893709 |
| *Sarcocystis venatoria* | KY973325−KY973326, KY973328−KY973329, KY973331 | KY973287, MT070689, MT070691, MT070693, MT070695 |
| *Toxoplasma gondii* | XR001974458 | MN077082 |
